# Behavioral Biases and Investment Decision-Making in the Indian Stock Market: The Moderating Role of Financial Literacy and Investor Experience.

**DOI:** 10.12688/f1000research.171289.2

**Published:** 2026-01-19

**Authors:** Narasaraju Divakara Reddy, B R Santosh, Ananda S, Guruprasad Desai

**Affiliations:** 1 Department of Commerce, Manipal Academy of Higher Education, Manipal, Karnataka, 560063, India; 2 Postgraduate Studies, Research & Innovation Department, College of Banking and Financial Studies, Ruwi, Muscat Governorate, Oman

**Keywords:** Investment Decisions; Behavioral Finance; Investor Experience; Financial Literacy.

## Abstract

**Background:**

Investment decision making is a critical aspect of financial planning. It involves allocating the financial resources to various investment avenues with an objective of generating future returns. Behavioral finance provides a theoretical framework for understanding psychological biases investment decision making which changes the assumptions of traditional finance. This study examines five important behavioral biases such as heuristics, prospect theory, emotions, market impact, and herding behavior on investment decision-making and portfolio management by considering investor experience and financial literacy as moderating factors.

**Methods:**

Primary data was collected from individual investors in the BSE and NSE using a structured questionnaire administered through a purposive random sampling. Resulting in 151 complete responses were obtained and were considered valid for the purpose of our study. PLS-SEM was used to test the proposed hypotheses as well as the moderating effect

**Results:**

Study finding indicates that heuristics have a positive and statistically significant effect on investment decisions, while prospect theory, emotions, market impact, and herding behavior showed no significant direct influence. The moderation analysis reveals that both investor experience and financial literacy significantly moderate the effects of emotions and market impact on investment decisions. However, their influence on heuristics, prospect theory, and herding behavior was statistically insignificant.

**Conclusions:**

The results of this study lead to several conclusions. In this present study only one behavioral bias heuristics (HU) demonstrated a significant direct influence on investment decisions. In contrast, other behavioral biases such as prospect theory (P), emotions (E), market impact (M), and herding behavior (HB) do not significantly affect investment decisions (p > 0.05). The moderating effect of investors’ experience and financial literacy investor behavior are minimal. The result underscores that improving financial education, skills, knowledge and gaining experience may help investors regulate emotional and trend-based decisions but may not be sufficient to address more instinctive cognitive biases. The significance of the study provides important implications for financial educators, advisors, policymakers and stock market authorities regarding the need for behaviorally informed investor training, decision-support systems, and informed advisory services to promote rational investment behavior.

## 1. Introduction

Investment decision making is a complex process that involves careful selection of financial assets to invest with the objective of achieving the future financial goal of maximizing returns by managing risks. Few investors make decisions based on judgement whereas others consider many other factors such as past stock performance, market trends, social influence and personal financial goals that direct them to make an appropriate decision ( Abideen et al., 2023). This decision-making process is influenced by several factors including financial literacy, investor experience, risk tolerance, locus of control and behavioral biases ( Abideen et al., 2023; Suresh, 2024).

Traditionally, investment decisions are analyzed based on classical finance theories such as Modern Portfolio Theory ( Markowitz, 1952) and the Efficient Market Hypothesis ( Fama, 1970) which assume that investors behave rationally, markets are efficient, and decisions are made based on expected returns. However, real-world investment practices and behaviors often deviate from those of traditional models. Investment decisions are more often influenced by intuition, biases, and psychological heuristics than objective analysis ( Charles & Kasilingam, 2015; Gupta & Bhardwaj, 2023; Makwana, 2024). This has led to the emergence of behavioral finance, a field that integrates cognitive psychology with financial theory to explain the irrational, biased, and emotional aspects of the investors influence on investment decision-making ( Tversky & Kahneman, 1974; Barberis & Thaler, 2003).

Behavioral biases play an important role in shaping investor decisions in financial markets. The study of behavioral biases among Indian investors on the Bombay Stock Exchange (BSE) and the National Stock Exchange (NSE) reveals the significant effects of these biases on investment decision-making. The key biases identified for this study are heuristics ( Tversky & Kahneman, 1974), prospect theory ( Barberis, Jin, & Wang, 2020), and emotions ( Tversky & Kahneman, 1991; Vuković & Pivac, 2024). Financial literacy and investor experience play a prominent role in moderating the impact of these biases on investment decisions ( Khan & Hassan, 2023; Mahmood et al., 2024; Wang & Zou, 2024). This study indicates that financial literacy is essential and does not significantly affect investment decisions on its own; rather, it interacts with behavioral biases to shape outcomes ( Amudha & Geetha, 2012) and years of investment experience can also influence susceptibility to these biases. More experienced investors may exhibit different behavioral patterns than inexperience, potentially mitigating the effects of biases ( Gherzi et al., 2014).

This study focuses on the individual investors in the Indian stock market. Stock markets play a very important role in the allocation of economic resources and economic development of a country ( Zuravicky, 2005). India has two premier stock markets: the Bombay Stock Exchange (BSE) and National Stock Exchange (NSE). BSE has played a major role in the Indian stock market, with a significant share of trading volumes ( Singh & Joshi, 2023; Shah, 2012). NSE was established to provide modernization and transparency in the Indian stock market. NSE consistently has a higher volume of trading than the BSE ( Goel et al., 2021; Krishnamurti & Lim, 2001). BSE has a larger retail investor, these retail investors are attracted to invest because of the historical significance and larger diversity of its listed companies. The majority of retail investors focus on long- term investment and follow conservative investment strategies ( Singh & Joshi, 2023; Shah, 2012). NSE has a higher proportion of institutional investors, including foreign institutional investors and domestic institutional investors. These investors are more active and engage in short- term trading strategies that use advanced trading systems and higher liquidity ( Goel et al., 2021; Krishnamurti & Lim, 2001).

In this context, this study assesses the behavioral biases of the individual investors of the Bombay stock exchange (BSE) and the National Stock Exchange (NSE) in the Indian stock markets. We also examined the moderating effects of financial literacy and investor experience.

## 2. Theoretical background and literature review

### 2.1 Heuristics

In investment decision-making, heuristics are frequently employed to simplify the evaluation of financial information, asset performance, and market trends. Most investors rely on recent price movements, familiar company names, or initial stock values to make quick judgements, often without conducting a thorough analysis ( Ricciardi & Simon, 2000).

Heuristics can also lead to systematic errors or cognitive biases, such as the anchoring effect, availability heuristics, framing effect, and confirmation bias ( Yasseri & Reher, 2019; Lyons & Kass-Hanna, 2021). These biases can cause investors to make suboptimal investment decisions, as they may rely on incomplete or biased information, fail to consider alternative perspectives, or exhibit overconfidence in their abilities ( Kumar & Goyal, 2015).

Dangol & Manandhar (2020) found that heuristics such as representativeness, availability, and anchoring are significantly associated with irrational investment decisions. According to Haritha (2023) revealed that investor sentiment mediates the influence of heuristics on stock selection ( Haritha, 2023). According to Parkash & Parkash (2024) found that institutional investors, such as fund managers, are prone to heuristics like overconfidence and anchoring, which skew asset selection and market timing decisions. According to Ahmad et al. (2025) empirically examined the influence of heuristic-driven cognitive biases among investors in emerging markets. They found that overconfidence, anchoring, and availability biases significantly disrupted rational investment behavior and led to poorly diversified portfolios.

Empirical studies have found that individual investors rely heavily on overconfidence and availability heuristics, which can lead to excessive trading and risk underestimation, particularly among young and active traders in markets such as Mumbai ( Kakkar & Hariharan, 2022).

H _1_. The use of heuristics has a significant impact on investment decision making process.

### 2.2 Prospect theory

The prospect theory was developed by Kahneman and Tversky. This explains how investors perceive gains and losses, emphasizing their loss aversion. Investment decision making highlights that investors prioritize avoiding losses over acquiring equivalent gains, influencing their financial choices and risk assessments ( Morais Rodopoulos & Silveira Júnior, 2024). It challenges traditional economic models, particularly the expected utility theory, by incorporating psychological factors into the decision-making process ( Reyna and Brainerd, 2012).

Prospect theory challenges the traditional notion of rationality by illustrating how individuals make decisions under risk, not based on objective outcomes, but on perceived gains and losses relative to a reference point ( Tversky & Kahneman, 1991). It challenges traditional economic models, particularly the expected utility theory, by incorporating psychological factors into the decision-making process ( Reyna and Brainerd, 2012).

An empirical analysis across U.S. and European markets found that Prospect Theory based portfolios consistently outperformed traditional mean-variance allocations. This highlights the practical value of behavioral considerations in portfolio construction ( Fortin & Hlouskova, 2024). Yadav & Daga (2023) found that biases derived from Prospect Theory particularly loss aversion and risk aversion significantly influence irrational investment decisions among Indian stock market participants. Khairunnisa (2024) applied Prospect Theory to study financial decision-making among university students. The results revealed strong behavioral tendencies such as loss aversion and temporal discounting, influencing both short- and long-term investment planning.

As a result, prospect theory provides a valuable framework for analyzing the psychological underpinnings of investor behavior, particularly in uncertain or volatile market conditions.

H _2_. Prospect Theory has a significant impact on investment decision making process.

### 2.3 Emotions

Emotions and psychological factors play a crucial role in shaping investment decisions and often influence financial choices in ways that traditional economic models fail to account. Emotions play a significant role in investment decision making, often leading investors to deviate from purely rational analyses ( Lerner et al., 2015).

Traditional finance theory often assumes that investors are rational actors who make decisions based on objective information and logical analysis. However, this assumption overlooks the powerful influence of emotions on human behavior ( Singh & Dhami, 2024). Emotions can shape how investors perceive risk, evaluate potential returns, and react to market fluctuations ( Brooks & Williams, 2022).

Furthermore, literature reviews reveal that external factors such as weather and religious observances can modulate investor mood and behavior, subtly shifting risk perception and investment appetite ( Annapurna & Basri, 2024). According to Sutejo et al. (2024) positive emotions significantly enhanced investment decisions, while negative emotions had mixed or insignificant effects. The study also revealed that financial risk tolerance mediates the influence of these emotions on portfolio choices. Theoretical work underscores those successful investors not only recognize their emotional states, but also regulate them to align with strategic objectives, merging affective awareness with cognitive rigor ( Das & Panja, 2020).

H _3_. Use of emotions has a significant impact on investment decision making process.

### 2.4 Market impact

In modern financial markets, investment decisions are increasingly influenced by external factors and real-time market dynamics. The term market impact broadly refers to the influence of market-related factors such as price trends, volatility, trading volume, economic indicators, and media coverage on investor behavior. Rather than relying solely on intrinsic valuations or fundamental analysis, investors often react to what is happening in the market, especially when faced with uncertainty or rapidly changing conditions ( Barberis & Thaler, 2003; Shiller, 2000).

Investors in established markets such as India actively consider macroeconomic conditions such as GDP growth, inflation, interest rates, and political stability when forming their investment strategies ( Varshini and Vinayalaxmi, 2024). Karimi & Nasieku (2024) examined the Nairobi Securities Exchange and revealed that behavioural biases intensify market volatility by encouraging reactionary trading patterns and premature selling or holding decisions. Zain Ui Abideen et al. (2023) revealed that herding, overconfidence, and loss aversion biases interact with market anomalies, amplifying price volatility and mispricing indicating that biases can destabilize markets unless actively managed .

Empirical research indicates that dynamic market variables, such as past stock performance, customer preferences, and general market information are central to shaping investor behavior, especially in developing financial markets such as Nepal, where youth participation is rapidly increasing ( Dhungana et al., 2023).

H _4_. Market factors have a significant influence the investment decision making process.

### 2.5 Herding

Herding behavior, the tendency of investors to follow the actions of a larger group, can drive market trends and create bubbles or crashes ( Kellard et al., 2016). This behavior is especially prominent in financial markets, where investors faced with uncertainty, time pressure, or informational ambiguity, and tend to follow the perceived consensus rather than act independently.

Herding behavior leads to suboptimal investment performance among individual investors ( Zafar et al., 2024). This imitation can be seen in both bullish and bearish market conditions which may lead to overpricing and speculative bubbles while in downturns, it may trigger panic selling and excessive pessimism ( Shiller, 2000; Devenow & Welch, 1996).

Chang, Cheng, and Khorana (2000) report significant herding effects in emerging markets, where informational asymmetry is higher and investor sophistication tends to be lower. In retail investing, herding has been linked to limited financial literacy, fear of missing out, and the growing influence of social media on investment sentiment ( Kliger & Levy, 2003).

H _5_. Herding has a significant impact on investment decision-making process.

### 2.6 Financial literacy and Investment decision

Behavioral biases are detrimental to investment decision making and recent studies have increasingly focused on the moderate effect of financial literacy. Financial literacy is defined as the ability to understand and apply basic financial concepts to financial decisions. It can significantly mitigate the adverse effects of behavioral biases ( Lusardi & Mitchell, 2014). A high level of financial literacy and skills help investors to analyze information critically, make informed decisions, and thus reduce reliance on heuristics that might lead to biased decisions ( Hirshleifer & Teoh, 2003) and better investment outcomes, including improved portfolio diversification and reduced impulsive trading behaviors ( Subnani & Todwal, 2024). However, the moderating effect of financial literacy may vary depending on the type of bias and investor gender, with some studies suggesting that financial literacy is more effective at moderating the impact of cognitive biases among male investors ( Adil et al., 2021).

In a study of millennial investors in Jakarta, financial literacy moderated the effect of overconfidence on investment decisions, indicating that well-informed investors are better equipped to avoid overestimating their decision-making abilities ( Nadhila et al., 2024). A similar study conducted in the Iraqi stock market, shows that financial literacy strengthened the predictive relationship between behavioral factors such as firm image, advocate recommendations, financial needs and investment outcomes, suggesting that informed investors are better at integrating diverse inputs into their investment decisions ( Abdulridha & Hussin, 2024).

In the Indian context, financial literacy is considerably low, particularly among young and less educated populations ( Lusardi & Mitchell, 2011). According to Sood and Jain (2021), increased financial education programs can enhance an individual’s understanding of investment products, thus empowering investors to counteract biases such as overconfidence and loss aversion. The authors argue that financial literacy serves as a buffer against psychological factors, enabling investors to remain rational and resulting in better portfolio performance.

Financially literate investors are more likely to conduct independent research, scrutinizing market trends rather than simply following market sentiments. Such investors demonstrate a stronger capacity to withstand social pressures inherent in investment environments ( Prakash & Gupta, 2021).

H _6_. Financial literacy moderates the relationship between behavioral biases in investment decisions among investors in the Indian stock markets.

### 2.7 Investor experience and Investment decision

Investor experience moderates the impact of behavioral biases on investment decision and portfolio management decisions. Novice investors tend to be more susceptible to biases, such as herding and anchoring, whereas experienced investors may utilize their insights to mitigate these effects ( Agarwal & Tiwari, 2018). Additionally, Malmendier, Pouzo & Vanasco (2020) found that while biases persist in all investor categories, the degree of their influence diminishes with increasing experience level.

Past research in the Indian context indicates that experienced investors are better equipped to deal with market volatility and may demonstrate greater resilience to emotional biases ( Agarwal & Tiwari, 2018). Investment experience helps individuals better recognize and manage their own behavioral patterns, leading to improved decision-making through diversified portfolios and realistic goal setting ( Atiq, 2024).

According to Chalissery (2023), financial literacy enhances this moderating effect, particularly in emerging markets such as India and Pakistan. Their findings show that while behavioral biases such as anchoring, overconfidence, and herding are prevalent, experienced investors with higher financial literacy are less influenced by them, suggesting a strong interplay between knowledge and experiential learning. Investor experience acts as a critical buffer against distortions caused by behavioral biases, helping investors make more informed and rational decisions.

H _7_. Investors’ experience moderates the relationship between behavioral biases in investment decisions among investors in the Indian stock markets.

**Figure 1.**
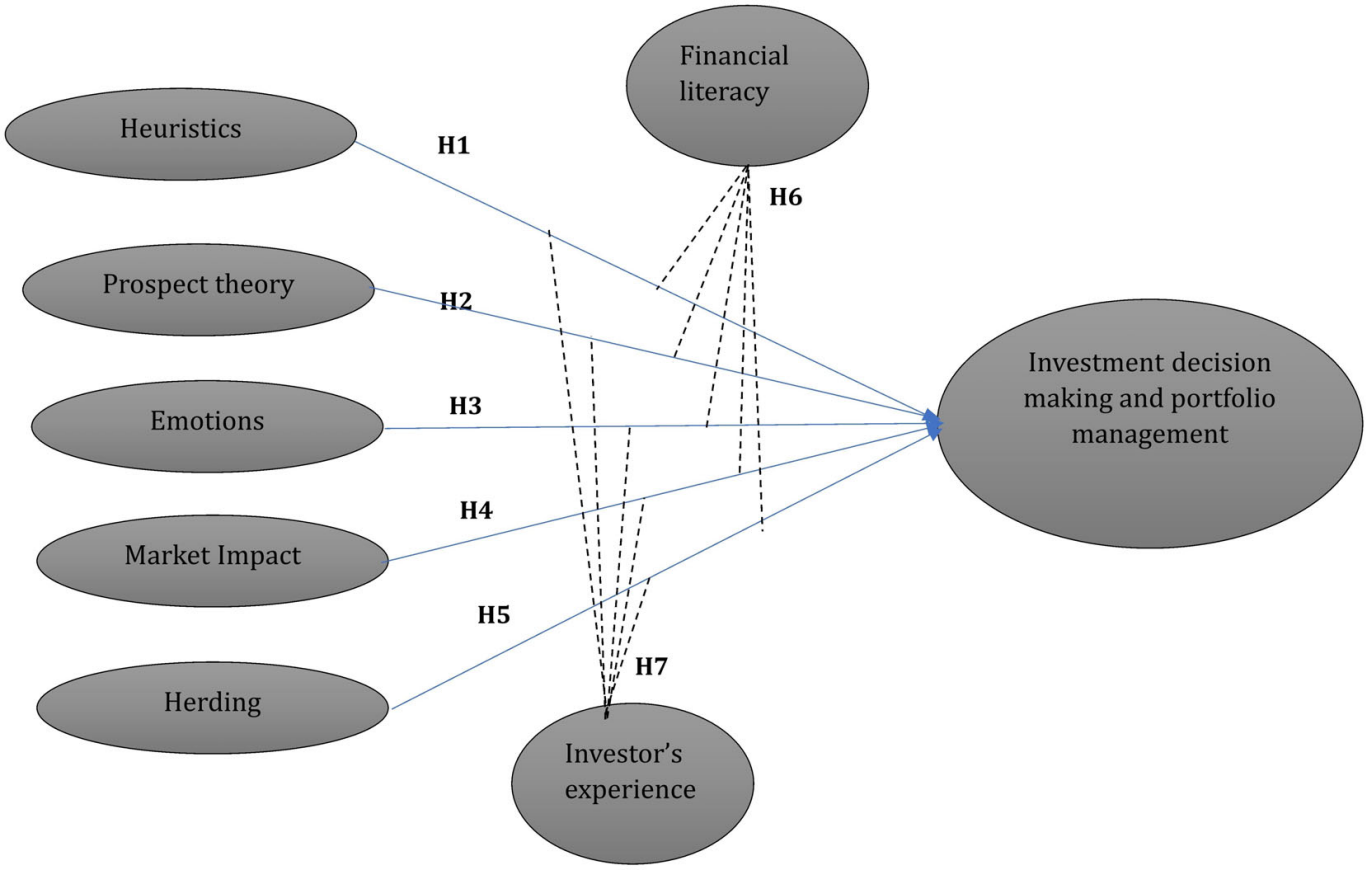
Conceptual framework. Source(s): Authors own creation.

**Based on the above literature the study develops the following conceptual framework ( Figure 1).**

## 3. Data and methodology

This study focuses on individual investors in the BSE and NSE stock exchanges in India. The data for the study were collected using purposive random sampling. A purposive random sampling approach allowed the researcher to identify target respondents who actively participate in investment activities, ensuring that the data reflected informed decision-making behaviors ( Etikan et al., 2016). The relatively smaller sample size is attributable to the sensitivity of investment-related data and participant reluctance to disclose personal financial information. At the same time, a random component was incorporated to minimize sampling bias and enhance representativeness. The survey approach was considered the most appropriate for this study because it was difficult to find respondents. The target population for this study was real investors on the BSE and NSE stock exchanges. Respondents were invited to fill out the Google Forms questionnaire and the link sent through email and WhatsApp messages. To increase participation, confidentiality of the data was assured.

The primary data were collected using a survey-based technique for the present research. As per this study’s objective, only a specific segment of the population was included. Therefore, the data have been collected “subjectively, but from a relevant segment of population” ( Sahi and Arora, 2012). Primarily, 350 individual investors were targeted, out of which 151 complete responses were obtained and were considered valid for the purpose of our study (Refer Table 1).

Based on previous research and literature on behavioral finance, five-point Likert scale questionnaires were designed to measure the variables ( Waweru et al., 2008; Charles and Kasilingam, 2015; Rekha, 2020). The questionnaire consists of 57 questions of which 7 demographic information, 5 questions measure investment decisions and portfolio management, 27 questions measure the influence of behavioral biases, and 18 questions to evaluate the moderating effect of financial literacy and investor experience.

SPSS and Smart PLS were used to analyze the data. SPSS was used to tabulate the responses of the respondents and refine the data of the variables data. PLS-SEM was used to test the hypotheses, as well as the moderating effect of financial literacy and investors experience on investment decisions.

During the empirical analysis, the study employed the compressive set of statistical tools such as descriptive statistics to summarise the demographic factors, correlation analysis used to assess the relationship among variables, Cronbach’s alpha used to evaluate the internal consistency and reliability of multi item scales, a simple regression test was conducted to test the hypotheses and determine the predictive power of the independent variable on investment decision making and subsequently utilized the empirical findings to examine the interrelations among various variables within the regression model.

## 4. Results and discussions

### 4.1 Descriptive analysis

Table 1 presents the summary statistics of the study’s respondents; majority of the respondents were male 65% and 35% were female. Among the respondents, A majority 45% were from the age group of 26-35 and 41% respondents were from the age group of 36-54 it indicates that investments are mainly focused on the economically active adults. There is a lack of interest in young investors and senior citizens. The finding indicates that nearly 64% of the respondents were postgraduates are the investors and 15% were PhD holders, which shows that majority of the respondents were highly educated sample. marital status of the 65% respondents was married and unmarried, or singles are 29%. Regarding to the employment status, more than half of the respondents 57% are employed in private sector, 17% in government sector, 15% were self-employed and remaining 11% have belonged to other categories. Occupation wise professions were the majority in the group 42%, followed by technical roles 15% and administrative and clerical jobs 14%. Finally, it shows the majority of the respondents 53% invest 10% of their income to investment, 25% of the respondents are investing 11%-20% of their income to investments and only 9% investors are aggressively investing their income.

**Table 1.** Descriptive analysis.

Description	Classification	Frequency	Percentage
Gender	Male	98	65
	Female	53	35
Age	Under 25	17	11
	26-35	68	45
	36-54	62	41
	55 & above	4	3
Educational level:	Secondary Education	4	3
	Under Graduate	27	18
	Post Graduate	97	64
	Doctorate	23	15
Marital Status	Single	44	29
	Married	103	65
	Divorced	3	2
	Widowed	1	1
Employment Status	Government employee	26	17
	Private sector employee	86	57
	Self-employed	23	15
	Others	16	11
Occupation	Management	18	12
	Professional	63	42
	Technical	23	15
	Administrative/Clerical	22	14
	Skilled labor	7	5
	Service industry	18	12
Annual investment	Up to 10%	80	53
	11% to 20%	38	25
	20% to 30%	19	13
	30% to 40%	14	9

Source(s): Authors’ own work, 2025.

### 4.2 Measurement model analysis

The reliability and validity of the study variables were evaluated using key metrics for reflective measurement models: outer loading, Average Variance extracted (AVE) composite reliability and Cronbach’s alpha ( Hair, 2010).

The factor loadings for the constructs varied as shown in Table 2. For heuristics, loadings ranged from 0.644 to 0.78. The prospect theory items had loadings between 0.631 and 0.772. Their emotions ranged from 0.616 to 0.78. The market impact ranged from 0.682 to 0.84. herding ranged from 0.615 to 0.877, investment decisions ranged from 0.737 to 0.807, Financial Literacy ranged from 0.731 to 0.817 and Investor Experience ranged from 0.766 to 0.846. The value of the constructs was greater than the threshold of 0.50. This suggests that each item adequately represents its respective construct ( Tang, 2024).

An AVE value of 0.5 or higher suggests adequate convergent validity, indicating that the indicators represent the underlying construct well ( Hair et al., 2019). The Table 2 shows that the AVE values for each construct are above the threshold of 0.5, indicating that all constructs are typically considered acceptable ( Hair et al., 2010) and indicates that the indicators used to measure the constructs are strongly related to the underlying concepts.

According to Hair et al. (2019) and Hair et al. (2021), a Composite reliability value between 0.70 and 0.90 is considered acceptable and indicates satisfactory internal consistency. Conversely, values below 0.70 are considered inadequate and reflect poor reliability, suggesting the need for model refinement. The Table 2 shows that CR values are for each construct are above the threshold of 0.7, indicating that all constructs are typically considered acceptable ( Hair et al., 2019) and this indicates that all constructs have high internal consistency suggesting that the items consistently measure the intended constructs ( Henseler, Ringle & Sarstedt 2009).

Cronbach’s alpha assesses how well a set of items measures a single underlying construct. The Table 2 shows that the values of Cronbach’s alpha for all the constructs were above 0.70, indicating good internal consistency and reliability ( Hair et al., 2010).

As all evaluation criteria have been met with acceptance, it can be concluded that there is greater support for reliability and validity.

**Table 2.** Composite reliability, AVE and outer loading.

Constructs	Items	Outer loading	AVE	Composite reliability	Cronbach’s alpha
Heuristics	HU 1	0.78	0.54	0.838	0.829
	HU 2	0.644			
	HU 3	0.759			
	HU 4	0.673			
	HU 5	0.773			
	HU 6	0.77			
Prospect theory	P 1	0.7	0.514	0.772	0.763
	P 2	0.738			
	P 3	0.735			
	P 4	0.631			
	P 5	0.772			
Emotions	E 1	0.78	0.533	0.729	0.708
	E 2	0.778			
	E 3	0.616			
	E 4	0.735			
Market Impact	M 1	0.84	0.598	0.792	0.776
	M 2	0.784			
	M 3	0.78			
	M 4	0.682			
Herding	HB 1	0.792	0.612	0.846	0.84
	HB 2	0.873			
	HB 3	0.877			
	HB 4	0.723			
	HB 5	0.615			
Investment Decision	ID 1	0.737	0.56	0.813	0.802
	ID 2	0.767			
	ID 3	0.783			
	ID 4	0.807			
Financial Literacy	FL 1	0.742	0.595	0.92	0.915
	FL 2	0.788			
	FL 3	0.798			
	FL 4	0.731			
	FL 5	0.817			
	FL 6	0.758			
	FL 7	0.767			
	FL 8	0.8			
	FL 9	0.736			
Investors Experience	IE 1	0.801	0.641	0.901	0.89
	IE 2	0.766			
	IE 3	0.792			
	IE 4	0.846			
	IE 5	0.8			
	IE 6	0.796			

Source(s): Authors’ own work, 2025.

The figure below shows the results of Confirmatory Factor Analysis by using SmartPLS software.

**Figure 2.**
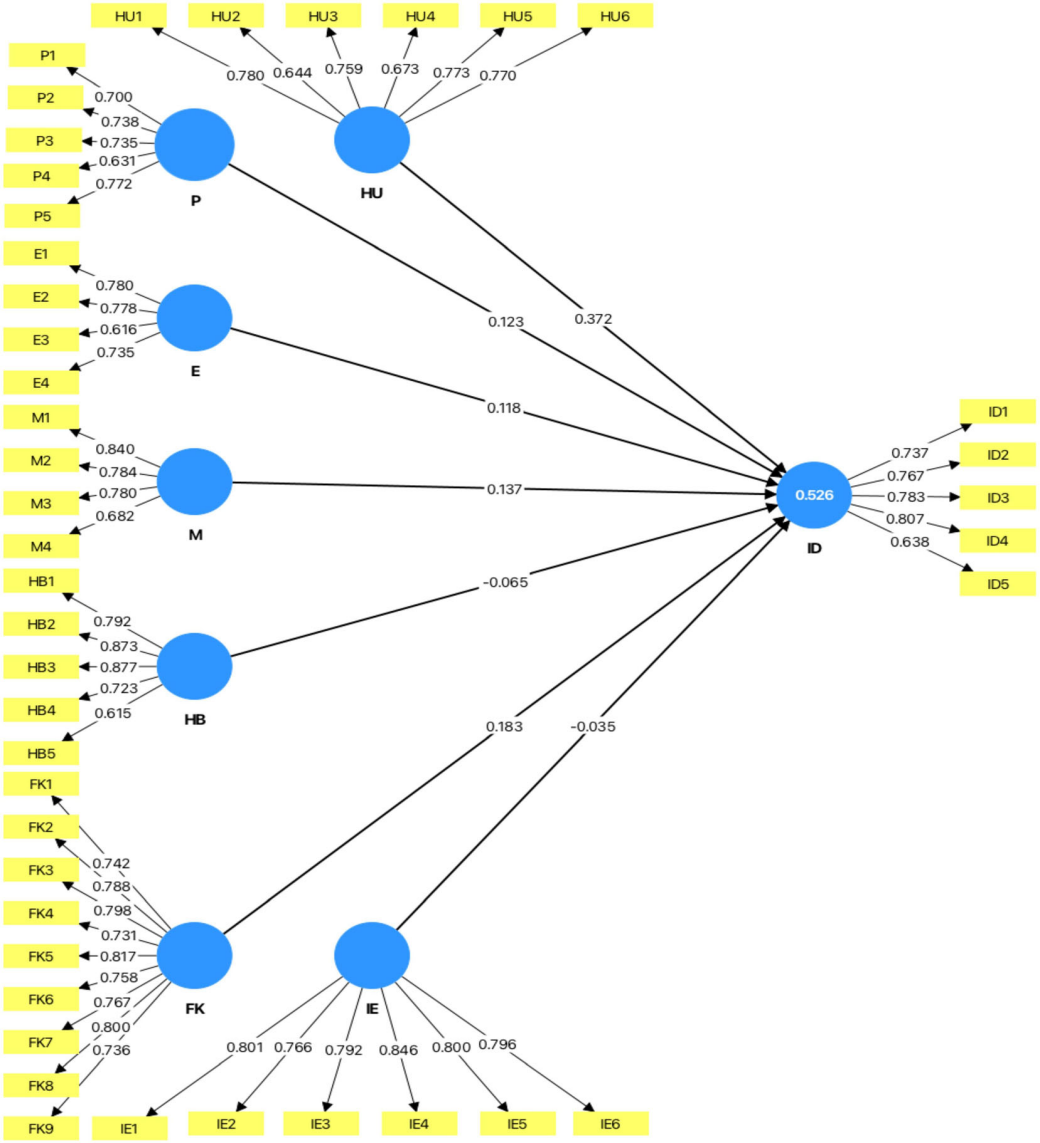
Measurement model for CFA. Sources: Smart PLS Output, Authors calculation.

Based on the Fornell–Larcker criterion, the discriminant validity of the construct was established when the square root of the Average Variance Extracted (AVE) for each construct exceeded the construct’s highest correlation with any other construct in the model ( Hair et al. 2019). As shown in Table 3, the square roots of AVE were greater than the inter-construct correlations indicating that discriminant validity is generally supported except between constructs E (emotions) and M (market impact), where the correlation (0.755) exceeded the square root of AVE for E (0.73) indicating that there is an overlap between these constructs and a lack of discriminant validity.

**Table 3.** Discriminant validity (Fornnel -Larcker criterion).

	E	FK	HB	HU	ID	IE	M	P
**E**	**0.73**							
**FK**	0.605	**0.771**						
**HB**	0.498	0.451	**0.782**					
**HU**	0.724	0.496	0.572	**0.735**				
**ID**	0.625	0.508	0.411	0.674	**0.748**			
**IE**	0.569	0.736	0.439	0.503	0.451	**0.801**		
**M**	0.755	0.49	0.497	0.703	0.599	0.508	**0.774**	
**P**	0.619	0.466	0.565	0.69	0.565	0.456	0.585	**0.717**

**Source(s):** Authors’ own work, 2025.

Emotions ( **E**), Financial Literacy ( **FK**), Herding ( **HB**), Heuristics ( **HU**), Investment Decision ( **ID**), Investors Experience ( **IE**), Market Impact ( **M**), Prospect theory **(P)**.

**Table 4.** HTMT.

	E	FK	HB	HU	ID	IE	M	P
**E**								
**FK**	0.73							
**HB**	0.64	0.462						
**HU**	0.948	0.565	0.674					
**ID**	0.807	0.578	0.464	0.814				
**IE**	0.687	0.812	0.452	0.56	0.507			
**M**	1.018	0.574	0.598	0.877	0.744	0.592		
**P**	0.83	0.553	0.701	0.871	0.716	0.542	0.759	

**Source(s):** Authors’ own work, 2025.

Emotions ( **E**), Financial Literacy ( **FK**), Herding ( **HB**), Heuristics ( **HU**), Investment Decision ( **ID**), Investors Experience ( **IE**), Market Impact ( **M**), Prospect theory **(P)**.

Furthermore, the discriminant validity of the constructs was established using the Heterotrait - Monotrait Ratio (HTMT) of the correlations. Discriminant validity is established when the HTMT values are below 0.85 for conceptually distinct constructs ( Hair et al., 2021). The HTMT ratio is a more sensitive method than the Fornell-Larcker criterion for detecting discriminant validity issues using variance-based structural equation modeling ( Henseler, Ringle, & Sarstedt, 2009; Hair et al., 2021). As shown in Table 4, most constructs met the threshold of less than 0.85, indicating discriminant validity. However, certain sub constructs HTMT values exceed the threshold of 0.85, such as the constructs of emotions (E) and heuristics (HU) at 0.948 and emotions (E) and market impact (M) at 1.018. The constructs with high cross-loadings and conceptual redundancy were carefully reviewed ( Sarstedt, Ringle, & Hair, 2020). Overlapping indicator within the emotions construct, heuristics (HU) and market impact was removed to improve construct distinctiveness. Following this refinement, the recalculated AVE and HTMT values met the recommended threshold, confirming that discriminant validity was established across all constructs in the final measurement model. These steps ensured that each construct was conceptually unique and adequately captured its theoretical dimension ( Farrell, 2010; Kline, 2016).

### 4.3 Structural model

After establishing the requirements of the measurement model, the structural model was used to test the direct effect of various constructs on investment decisions measured through path coefficient analysis using PLS-SEM. The results of the direct effect of H1 to H5 hypotheses are presented in Table 5, the results of acceptance or rejection of the hypotheses mentioned in column 7 of Table 5. T statistics and P values were used to measure the significance of the hypotheses. The evaluation was based on the significance of t-statistics and p-values as recommended by Hair et al. (2021). According to Hair et al. (2021), the t-statistic must exceed 1.645 by assuming that a one-tailed test at the 5% significance level and the corresponding p-value should be less than 0.05. These thresholds provide sufficient evidence to confirm the statistical significance of hypothesized relationships ( Hair et al., 2021; Sarstedt et al., 2022; Henseler et al., 2009).

**Table 5.** Hypothesis testing.

H No.	Path	Original sample	Standard deviation	T statistics	P values	Result
H1	HU -> ID	0.372	0.112	3.324	0	Accept
H2	P -> ID	0.123	0.105	1.168	0.121	Reject
H3	E -> ID	0.118	0.132	0.893	0.186	Reject
H4	M -> ID	0.137	0.106	1.297	0.097	Reject
H5	HB -> ID	-0.065	0.074	0.878	0.19	Reject

Source(s): Authors’ own work, 2025.

The empirical results are presented in Table 6. The p-value for the heuristics (HU) is 0.000, which is less than the conventional significance level of 0.05, and the t-statistic is 3.324, which exceeds the threshold of 1.645. These results indicated that the hypothesis (HU → ID) was accepted. This finding suggests a statistically significant and positive relationship between heuristics and investment decisions.

The p-value for prospect theory (P) is 0.121, which is higher than the conventional significance level of 0.05, The t-statistic is 1.168, which is below the significance threshold of 1.645. These results indicate that hypothesis (P → ID) is not supported. This finding indicates that there is no statistically significant relationship between prospect theory and investment decision.

The p-value for emotions (E) is 0.186, which is higher than the conventional significance level of 0.05, and the t-statistic is 0.893, which is below the significance threshold of 1.645. These results indicate that the hypothesis (E -> ID) is not accepted. This finding indicates that there is no statistically significant relationship between emotions and investment decisions.

The p-value for Market Impact (M) is 0.097, which is higher than the conventional significance level of 0.05, and the t-statistic is 1.297, which is below the significance threshold of 1.645. These results indicate that hypothesis (M -> ID) is not accepted. This indicates that there is no statistically significant relationship between the market impact and investment decision.

The p-value for herding (H) is 0.19, which is higher than the conventional significance level of 0.05, and the t-statistic is 0.878, which is below the significance threshold of 1.645. These results indicate that hypothesis (M -> ID) is not accepted. This finding indicates that there is no statistically significant relationship between herding and investment decisions.

**Table 6.** Hypothesis testing-Moderation effect of investors experience.

H No.		Original sample	Standard deviation	T statistics	P values	Result
	IE x E -> ID	0.331	0.131	2.524	0.006	Accept
	IE x FK -> ID	0.023	0.083	0.274	0.392	Reject
	IE x HB -> ID	-0.148	0.112	1.316	0.094	Reject
	IE x HU -> ID	0.003	0.125	0.025	0.49	Reject
	IE x M -> ID	-0.291	0.114	2.542	0.006	Accept
	IE x P -> ID	-0.067	0.103	0.654	0.257	Reject

Source(s): Authors’ own work, 2025

### 4.4 Moderating role of investors experience between behavioral biases and investment decision making

The study used a Moderated Multiple Regression revealed that Investor Experience significantly moderates the relationship between the two behavioral biases and investment decisions such as Emotions and Market Impact. The interaction between Investor Experience and Emotions (IE × E) shows a positive and statistically significant effect (β = 0.331, t = 2.524, p = 0.006). Suggesting that market fluctuations are less likely to influence emotional biases provides a better investment decision when investors have more experience. Similarly, the interaction terms between Investor Experience and Market Impact (IE × M) are negative and significant (β = -0.291, t = 2.542, p = 0.006). Suggesting that the influence of market impact biases on the investment decisions of experienced investors is less likely to be influenced by market fluctuations. However, the moderating effects of Investor Experience on herding behavior, heuristics, and prospect theory were found to be statistically insignificant. The evaluation was based on the criteria recommended by Hair et al. (2021), where a t-statistic greater than 1.645 and a p-value less than 0.05 indicate statistical significance. These findings that investor experience does not significantly influence these factors in investment decisions.

The moderation analysis examined whether investor experience influenced the relationship between behavioral biases and investment decisions. A significant and positive interaction indicates that the strength of the relationship between emotional bias and investment decisions increases as investor experience grows. Conversely, a significant and negative interaction suggests that the influence of market-related information on investment decisions weakens with greater investor experience. These results express that while experienced investors may become more confident in their emotions when making decisions, they are less influenced by external market signals.

Overall, these results suggest that investor experience does not uniformly impact behavioral biases, but rather redesign their influence by strengthening emotional self-regulation while reducing dependence on external market cues.

### 4.5 Moderating role of financial literacy between behavioral biases and investment decision making

Table 7 shows the results of the moderating role of financial literacy between behavioral biases and investment decision making. Moderation analysis revealed that financial literacy significantly influences the relationship between behavioral biases and investment decision-making. Out of the six hypothesized moderation effects, two are statistically significant, indicating that financial literacy influences the relationship between certain behavioral biases and investment decisions.

Specifically, financial literacy significantly moderates the effect of emotions (β = 0.334, t = 2.376, p = 0.009) and the market impact (β = -0.322, p = 0.003) on investment decisions. Financially literate investors are in a better position to critically evaluate market factors and avoid herd-behavior or cognitive based errors ( Baker et al., 2019).

However, the moderation analysis also revealed that financial literacy has not significant moderation effect of herding bias, heuristic, and prospect theory components on investment outcomes because interaction terms did not meet the threshold for significance (p > 0.05). The above results clearly show that financial literacy is not completely influential on all behavioral constructs but rather shows a conditional moderation.

**Table 7.** Hypothesis testing-Moderation effect of financial literacy.

H No.		Original sample	Standard deviation	T statistics	P values	Result
	FK x E -> ID	0.334	0.141	2.376	0.009	Accept
	FK x HB -> ID	0.085	0.108	0.786	0.216	Reject
	FK x HU -> ID	-0.145	0.118	1.235	0.108	Reject
	FK x IE -> ID	-0.028	0.066	0.419	0.338	Reject
	FK x M -> ID	-0.322	0.119	2.703	0.003	Accept
	FK x P -> ID	0.001	0.102	0.011	0.496	Reject

Source(s): Authors’ own work.

## 5. Conclusion

The study investigated the influence of behavioral biases on investment decision among individual investors and investment decisions when individual investors choose to invest under the effect of moderating factors, such as financial literacy and investors experience.

The results of this study lead to several conclusions. In this present study only one behavioral bias heuristics (HU) demonstrated a significant direct influence on investment decisions. This supports the findings of ( Waweru et al., 2008), who highlight that investors more often follow the simplified rule of thumb, especially under uncertainty. The strong relationship between heuristics and investment decision making indicates that there is a continued relevance of representativeness and availability in investor behavior, lending support to H1.

In contrast, other behavioral biases such as prospect theory (P), emotions (E), market impact (M), and herding behavior (HB) do not significantly affect investment decisions because they are below the threshold (p > 0.05), leading to the rejection of the hypotheses H2, H3, H4, and H5, which is consistent with previous literature on behavioral finance ( Riyazahmed & Saravanaraj, 2016). The insignificance of these biases may be attributed to contextual factors such as investors access to financial information, enhanced analytical tools, and increased investor sophistication, which potentially minimize the direct influence of prospect theory, market impact, herding behavior and emotional biases on decision-making.

Emotions do not influence investment decision making, several past studies comparing individual and institutional investors have found that emotional biases affect individual investors’ performance but not their decision-making processes ( Shafqat, 2024; Wijaya & Elgeka, 2024; Sutejo et al., 2024). This suggests that emotions may operate indirectly or be regulated by investors experience and learning, rather than exerting a direct behavioral influence on investment choices.

The impact of market factors on investment decisions is also rejected. These results imply that the market factors do not directly affect investment decisions. As market factors including short-term volatility, volume fluctuations and price trends do not significantly influence investors decision making. The extant literature also indicates that market impact alone does not strongly influence investment decisions ( Riyazahmed & Saravanaraj, 2016; Dumohar et al., 2022; Murhadi et al., 2024). This finding revels a shift toward more informed and analysis-driven investment behavior, particularly in markets having a greater transparency and regulatory observations.

Significant effect of herding behavior was rejected. Several studies have found that herding bias does not significantly influence investment decisions. Hu et al. (2021), suggests that herding is not a universal driver of investment decisions ( Zahro & Singgih, 2024). This may indicate that individual investors increasingly depending on their personal judgment and use financial knowledge rather than following collective market behavior.

This study provides strong evidence that prospect theory (H2), emotions (H3), market factors (H4), and herding behavior (H5) do not significantly influence investment decision-making, which leads to the formal rejection of these hypotheses. These findings identifies that the influence of these biases may be conditional, indirect, or moderated by investor-specific characteristics.

The moderating role of investors experiences between various behavioral biases and investment decision making. Investors experience positive and significantly moderate relationships between emotions → investment decisions and market impact → investment decisions. These findings suggest that investors’ experiences can lead to better emotional impulses in a better way, transforming them into more informed judgements rather than radical decisions. Our findings are consistent with empirical evidence in the literature that investor experience plays a crucial role in shaping investment decisions. With greater investment experience, investors are more likely to exhibit rational behavior, even though they may still be influenced by emotional biases ( Shafqat, 2024; Sharma & Prajapati 2024; Spytska, 2024; Utama et al., 2024). Similarly, the findings of the study show that other behavioral biases such as heuristics (HU), Prospect Theory (P) and herding behavior (HB) were found to have a positive and statistically insignificant moderating effect on investors experience between behavioral biases and investment decisions.

The moderating effect of financial literacy on various behavioral biases and investment decision making. Financial literacy has a significant moderating effect on the two behavioral biases of emotional biases and market impact. The study confirms that the role of emotional biases and market impact always fosters deliberate and informed investment decision-making moderated by financial literacy. The results of other behavioral biases such as heuristics (HU), Prospect Theory (P), emotions (E), and herding behavior (HB) were found to have a positive and statistically insignificant moderating effect of financial literacy between behavioral biases and investment decisions. The study findings are consistent with those of Adil et al. (2021) who found that overconfidence, risk aversion, herding, and disposition of behavioral biases are positive and partially significant only for overconfidence in males. According to the study conducted by Mahmood et al. (2024) overconfidence, herding, risk aversion, and disposition of behavioral biases is positive and statistically insignificant.

These findings suggest that these biases may persist regardless of investors’ experience and financial knowledge and that their moderating effect on investor behavior may be minimal or context-dependent within our targeted population. These insignificant results can be attributed to increased investor awareness, a rise in financial technology, improved regulatory frameworks, diversification in financial strategies, and market maturity.

It is suggested to enhance financial literacy programs for investors and promote awareness of behavioral biases to improve investment decisions and market stability in the Indian stock market. Financial educators and policymakers should focus on creating innovative market opportunities and educational initiatives to promote informed investment decisions, ultimately leading to more rational market behavior. Additionally, investors should gain experience through practical engagement to navigate biases better.

## Ethical approval

This is an original work of authors.

## Data Availability

Figshare: **Behavioral Biases and Investment Decision-Making in the Indian Stock Market**. https://doi.org/10.6084/m9.figshare.30235192 ( Desai, 2025). Data are available under the terms of the Creative Commons Zero “No rights reserved” data waiver (CC0 1.0 Public domain dedication).
