# Supplementary material for: Behavioral Biases and Investment Decision-Making in the Indian Stock Market: The Moderating Role of Financial Literacy and Investor Experience
Source: F1000Res. 2026 Jan 19;14:1283. Originally published 2025 Nov 19. [Version 2] doi: 10.12688/f1000research.171289.2 (PMC12824484; doi:10.12688/f1000research.171289.2)
Supplement: Peer review history

# Reviewer report: Reviewer response for version 2

**DOI:** 10.5256/f1000research.194780.r451207

**Published:** 2026-01-21

**Authors:** Dr. ASHIQUE ALI K A, Dr. Muhammed Safwan K K

**Affiliations:** 1 School of Law, Narsee Monjee Institute of Management Studies University - Hyderabad Campus, Jadcherla, Telangana, India; 2 Department of Commerce, Kuniya College of Arts and Science, Kasargod, Kerela, India

The study investigates how behavioural biases shape one's investment decision by analysing the data collected from stock market investors in India. Further, they analyse the moderating effect of both financial literacy and investor experience on this relationship. The authors have diligently revised the manuscript to address the suggested changes, and it has led to a substantial improvement in the manuscript’s structure, clarity, and overall presentation. Some grammatical errors are still present and may be addressed through careful proofreading. Otherwise, the authors' responses to the comments are satisfactory.

Is the work clearly and accurately presented and does it cite the current literature?

Yes

If applicable, is the statistical analysis and its interpretation appropriate?

Yes

Are all the source data underlying the results available to ensure full reproducibility?

Yes

Is the study design appropriate and is the work technically sound?

Partly

Are the conclusions drawn adequately supported by the results?

Partly

Are sufficient details of methods and analysis provided to allow replication by others?

Partly

Reviewer Expertise:

Our research experience includes technology acceptance, mobile payment systems, financial literacy, and behavioural finance.

We confirm that we have read this submission and believe that we have an appropriate level of expertise to confirm that it is of an acceptable scientific standard.

# Reviewer report: Reviewer response for version 1

**DOI:** 10.5256/f1000research.188884.r435274

**Published:** 2025-12-30

**Authors:** Dr. ASHIQUE ALI K A, Dr. Muhammed Safwan K K

**Affiliations:** 1 School of Law, Narsee Monjee Institute of Management Studies University - Hyderabad Campus, Jadcherla, Telangana, India; 2 Department of Commerce, Kuniya College of Arts and Science, Kasargod, Kerela, India

**Review Report**

*Summary of the Article*

 The study investigates how behavioural biases shape one's investment decision by analysing the data collected from stock market investors in India. Further they analyse the moderating effect of both financial literacy and investor experience on this relationship. They collect primary data from 151 respondents who are investing in stock market through NSE or BSE in India. The analysis was done using PLS-SEM. Subsequently, they find that heuristics have a significant positive impact on investment decision, while other biases show no effect. The results contradict with extant literature as they find insignificant effect of behavioural bias on investment decision. The moderating analysis reveals significant moderate effect of both the moderator variables, showing the importance of financial education in stock market investment. 

**1. Introduction**

 The introduction section may be extended to provide a detailed background for the study. Particularly, the section may shed light on why India was chosen as the research setting and why the specific moderators were selected. This would help readers better understand the research context.

**2. Theoretical Background and Literature Review**

 The theoretical background and literature review section shall justify the variable selection and hypotheses. This article provides a comprehensive review of the existing literature. However, the authors may consider starting the section with the dependent variable, so that readers understand the operational definition of investment decisions used in the study. It is important to know whether the authors emphasise measuring biased investment decisions or rational investment decisions. Also, the phrase ‘portfolio management’ may be removed from the name of the dependent variable. The hypotheses could be refined for precision and clarity. Overall, the study has considered only five behavioural biases, for which justification is needed. The choice of biases should be based on extant literature and theoretical background.

 A repetition is found on Page 4 under the heading 2.2 (Prospect Theory), and some open-ended citations appear to be wrongly put here.

*For e.g., “According to Haritha (2023) revealed that investor sentiment mediates the influence of heuristics on stock selection (Haritha, 2023).”*

 The sentence should have been in either of the following formats:

 a) *According to Haritha (2023), the investor sentiment mediates the influence of heuristics on stock selection*

*b) The investor sentiment mediates the influence of heuristics on stock selection (Haritha, 2023)*

**3. Methodology**

 The choice of PLS-SEM is appropriate; however, the choice may be justified using the arguments presented by Hair et al. (2018)

 It is understandable why there is a lower sample size, given the inhibition among people when it comes to talking about their investment behaviour. The authors may highlight the rationale behind the choice of purposive random sampling.

**4. Analysis and Results**

 The analysis protocol has been correctly followed with respect to PLS-SEM. However, there are some issues.

- Section 4.2 may be renamed as ‘Measurement Model Analysis’ or ‘Reliability and Validity Analysis’.
- There are some grammatical errors in the manuscript that need to be addressed, particularly in the results section. The manuscript needs a proofreading. E.g. *“The reliability and validity of the variables used in this study were evaluated using outer loadings, Average Variance Extracted (AVE), composite reliability, and Cronbach’s alpha are key metrics used to evaluate reflective measurement models”* should have been written as *"The reliability and validity of the study variables were evaluated using key metrics for reflective measurement models: outer loadings, Average Variance Extracted (AVE), composite reliability, and Cronbach’s alpha."*
- Section 4.3 is Cronbach’s Alpha, which has already been included in the title of Section 4.2. It should not be a separate section, and it shall be discussed along with composite reliability.
- An issue with discriminant validity has been reported, but no remedial actions have been taken. This needs to be resolved.
- The interpretation regarding the moderation effect needs to be revised. Significant and positive interaction means that the effect of emotions on investment decisions increases as the investor's experience increases. A significant and negative interaction indicates that the effect of market impact on investment decisions decreases as investor experience increases.

**5. Conclusion**

 The conclusion may be improved by discussing the probable reasons for both significant results and insignificant results. Having insignificant results needs to be thoroughly looked into. The authors need to clearly state the reasons for such results. The theoretical and practical implications of the study shall be elaborated. 

Is the work clearly and accurately presented and does it cite the current literature?

Yes

If applicable, is the statistical analysis and its interpretation appropriate?

Yes

Are all the source data underlying the results available to ensure full reproducibility?

Yes

Is the study design appropriate and is the work technically sound?

Partly

Are the conclusions drawn adequately supported by the results?

Partly

Are sufficient details of methods and analysis provided to allow replication by others?

Partly

Reviewer Expertise:

Our research experience includes technology acceptance, mobile payment systems, financial literacy, and behavioural finance.

We confirm that we have read this submission and believe that we have an appropriate level of expertise to confirm that it is of an acceptable scientific standard, however we have significant reservations, as outlined above.

# Response

**Published:** 2026-01-03

**Authors:** Divakara Reddy Narasaraju

**Affiliations:** Department of Commerce, Manipal Academy of Higher Education - Bengaluru Campus, Bengaluru, Karnataka, India

**1. The introduction section may be extended to provide a detailed background for the study. Particularly, the section may shed light on why India was chosen as the research setting and why the specific moderators were selected. This would help readers better understand the research context.**

**Response:**

 Thank you for the valuable feedback-

 Behavioral biases play an important role in shaping investor decisions in financial markets. The study of behavioral biases among Indian investors on the Bombay Stock Exchange (BSE) and the National Stock Exchange (NSE) reveals the significant effects of these biases on investment decision-making. The key biases identified for this study are heuristics (Tversky & Kahneman, 1974), prospect theory (Barberis, Jin, & Wang, 2020), and emotions (Tversky&Kahneman,1991) (Vuković & Pivac, 2024). Financial literacy and investor experience play a prominent role in moderating the impact of these biases on investment decisions (Khan & Hassan, 2023) (Mahmood et al., 2024) (Wang & Zou, 2024). This study indicates that financial literacy is essential and does not significantly affect investment decisions on its own; rather, it interacts with behavioral biases to shape outcomes (Amudha et al., 2012) and years of investment experience can also influence susceptibility to these biases. More experienced investors may exhibit different behavioral patterns than inexperience, potentially mitigating the effects of biases (Gherzi et al. 2014)

 This study focuses on the individual investors in the Indian stock market. Stock markets play a very important role in the allocation of economic resources and economic development of a country. (Zuravicky, 2005). India has two premier stock markets: the Bombay Stock Exchange (BSE) and National Stock Exchange (NSE). BSE has play a major role in the Indian stock market, with a significant share of trading volumes. (Singh & Joshi, 2023) (Shah, 2012).  NSE was established to provide modernization and transparency in the Indian stock market. NSE consistently has a higher volume of trading than the BSE. (Goel et al., 2021) (Krishnamurti & Lim, 2001). BSE has a larger retail investor, these retail investors are attracted to invest because of the historical significance and larger diversity of its listed companies. The majority of retail investors focus on long- term investment and follow conservative investment strategies (Singh & Joshi, 2023) (Shah, 2012).  NSE has a higher proportion of institutional investors, including foreign institutional investors and domestic institutional investors. These investors are more active and engage in short- term trading strategies that use advanced trading systems and higher liquidity. (Goel et al., 2021) (Krishnamurti & Lim, 2001).

 Vuković, M. and Pivac, S. (2024), "The impact of behavioral factors on investment decisions and investment performance in Croatian stock market", Managerial Finance, Vol. 50 No. 2, pp. 349-366. https://doi.org/10.1108/MF-01-2023-0068

 Khan, S., & Hassan, N. U. (2023). *Unlocking the investment puzzle: The influence of behavioral biases & moderating role of financial literacy*. Journal of Social Research Development, 4(2), 433–444. https://doi.org/10.53664/jsrd/04-02-2023-17-433-444

 Amudha, K., & Geetha, C. (2012). Behavioral factors influencing investment decisions of small investors. International Journal of Marketing, Financial Services & Management Research, 1(6), 45–59.

 Zuravicky, O. (2005). Behavioral finance and investor decision-making. Journal of Wealth Management, 8(3), 16–24. https://doi.org/10.3905/jwm.2005.470607

 Gherzi, S., Egan, D., Stewart, N., Haisley, E., & Ayton, P. (2014). **The more you pay, the more you buy: Price cues and purchase behavior**. *Journal of Economic Psychology*, **44**, 152–165.

 https://doi.org/10.1016/j.joep.2014.05.002

 Shah, A. (2012). India’s financial markets: An insider’s guide to how the markets work. Oxford University Press.

 Singh, S., & Joshi, M. (2023). Retail investor behavior and stock market participation in India. Journal of Asian Finance, Economics and Business, 10(1), 311–322.

https://doi.org/10.13106/jafeb.2023.vol10.no1.0311

 Krishnamurti, C., & Lim, K. (2001). **Institutional investors and stock market efficiency: Evidence from India**. *Journal of Multinational Financial Management*, **11**(1–2), 23–38.

https://doi.org/10.1016/S1042-444X(00)00045-2

 Goel, S., Seth, R., & Gupta, R. (2021). Trading volume, liquidity, and market efficiency: Evidence from BSE and NSE. Global Business Review, 22(5), 1281–1297.

 https://doi.org/10.1177/0972150919873182

**2. Theoretical Background and Literature Review**

**Response:**

 As suggested, revisions were made to the literature review section, and the hypotheses were refined for clarity by removing portfolio management, since it was not included as a study variable.

**3. Methodology**

**Response:**

 The data for the study were collected using purposive random sampling technique. A purposive random sampling approach allowed the researcher to identify target respondents who actively participate in investment activities, ensuring that the data reflected informed decision-making behaviors (Etikan et al., 2016). The relatively smaller sample size is attributable to the sensitivity of investment-related data and participant reluctance to disclose personal financial information. At the same time, a random component was incorporated to minimize sampling bias and enhance representativeness. Respondents were invited to fill out the Google Forms questionnaire and the link sent through email and WhatsApp messages. To increase participation, confidentiality of the data was assured.

 Etikan, I., Musa, S. A., & Alkassim, R. S. (2016). *Comparison of convenience sampling and purposive sampling.* *American Journal of Theoretical and Applied Statistics, 5*(1), 1–4.

**4. Analysis and Results**

**Response: **

1. 4.2 Replaced as “Measurement Model Analysis”
2. Section 4.3 has merged with 4.2.
3. The following are the “The interpretation regarding the moderation effect needs to be revised”.

The moderation analysis examined whether investor experience influenced the relationship between behavioral biases and investment decisions. A significant and positive interaction indicates that the strength of the relationship between emotional bias and investment decisions increases as investor experience grows. Conversely, a significant and negative interaction suggests that the influence of market-related information on investment decisions weakens with greater investor experience. These results express that while experienced investors may become more confident in their emotions when making decisions, they are less influenced by external market signals.

 constructs with high cross-loadings and conceptual redundancy were carefully reviewed (Sarstedt, Ringle, & Hair, 2020).  Overlapping indicator within the emotions construct, heuristics (HU) and market impact was removed to improve construct distinctiveness.  Following this refinement, the recalculated AVE and HTMT values met the recommended threshold, confirming that discriminant validity was established across all constructs in the final measurement model. These steps ensured that each construct was conceptually unique and adequately captured its theoretical dimension (Farrell, 2010; Kline, 2016).

 Kline, R. B. (2016). *Principles and Practice of Structural Equation Modeling* (4th ed.). Guilford Press.

 Farrell, A. M. (2010). *Insufficient discriminant validity: A comment on Bove, Pervan, Beatty, and Shiu (2009).* *Journal of Business Research, 63*(3), 324–327.

 Sarstedt, M., Ringle, C. M., & Hair, J. F. (2020). *Partial Least Squares Structural Equation Modeling.* In *Handbook of Market Research* (pp. 1–47). Springer.

**5. Conclusion**

**Response:** the following points added in the conclusion section

 The insignificance of these biases may be attributed to contextual factors such as investors access to financial information, enhanced analytical tools, and increased investor sophistication, which potentially minimize the direct influence of prospect theory, market impact, herding behavior and emotional biases on decision-making.

 This suggests that emotions may operate indirectly or be regulated by investors experience and learning, rather than exerting a direct behavioral influence on investment choices.

 This finding revels a shift toward more informed and analysis-driven investment behavior, particularly in markets having a greater transparency and regulatory observations.

 This may indicate that individual investors increasingly depending on their personal judgment and use financial knowledge rather than following collective market behavior.

 these findings identifies that the influence of these biases may be conditional, indirect, or moderated by investor-specific characteristics.

 We are grateful to the reviewers for their valuable and insightful comments, which have substantially improved the clarity and readability of the manuscript.

# Reviewer report: Reviewer response for version 1

**DOI:** 10.5256/f1000research.188884.r435273

**Published:** 2025-12-27

**Authors:** Dr. Priya Makhija

**Affiliations:** 1 Center for Management Studies, Jain University, Bengaluru, Karnataka, India

The study provides valuable insights by establishing heuristics as the only behavioral bias with a significant direct influence on investment decisions. The non-significance of prospect theory, emotions, market impact, and herding is well-explained and aligns with existing behavioral finance literature. The moderation results are meaningful, showing that investor experience and financial literacy strengthen decision-making specifically in the context of emotional and market-related biases.The discussion effectively links findings to broader trends such as increased investor awareness, fintech adoption, and market maturity. The study offers practical implications for policymakers and investors, though future research could explore additional behavioral variables and demographic moderating factors.

Is the work clearly and accurately presented and does it cite the current literature?

Yes

If applicable, is the statistical analysis and its interpretation appropriate?

Yes

Are all the source data underlying the results available to ensure full reproducibility?

Yes

Is the study design appropriate and is the work technically sound?

Yes

Are the conclusions drawn adequately supported by the results?

Yes

Are sufficient details of methods and analysis provided to allow replication by others?

Yes

Reviewer Expertise:

Finance, FinTech, CBDC, Indian Stock Market

I confirm that I have read this submission and believe that I have an appropriate level of expertise to confirm that it is of an acceptable scientific standard.
